# Pollination by sexual deception via pro‐pheromone mimicry?

**DOI:** 10.1111/nph.70131

**Published:** 2025-04-10

**Authors:** Ryan D. Phillips, Seeger van Kints, Ben Ong, Alyssa M. Weinstein, Rod Peakall, Gavin R. Flematti, Björn Bohman

**Affiliations:** ^1^ Department of Ecological, Plant and Animal Sciences La Trobe University Melbourne Vic 3086 Australia; ^2^ Research School of Biology Australian National University Canberra ACT 2600 Australia; ^3^ Royal Botanic Gardens Victoria Cranbourne Vic 3977 Australia; ^4^ School of Molecular Sciences University of Western Australia Perth 6009 WA Australia; ^5^ Tasmanian Institute of Agriculture University of Tasmania Hobart 7005 Tas Australia; ^6^ Department of Plant Protection Biology Swedish University of Agriculture 23422 Lomma Sweden

**Keywords:** chemical ecology, Ichneumonid wasp, orchid, pollination, pro‐pheromone mimicry, sexual deception

## Disclaimer

The New Phytologist Foundation remains neutral with regard to jurisdictional claims in maps and in any institutional affiliations.

## Introduction

Most plant species worldwide depend on insects for pollination (Ollerton *et al*., 2011), with volatile organic compounds being pivotal for mediating pollinator attraction in many of these plants (Raguso, 2008; Dötterl & Gershenzon, 2023). Among plants, orchids are exceptional in their extraordinary range of pollinators, pollination strategies, and floral volatiles (Ackerman *et al*., 2023; Perkins *et al*., 2023). One of the most remarkable pollination strategies is that of sexual deception, where the flower imitates female insects to attract male pollinators, with sex pheromone mimicry typically being key to pollinator attraction (Schiestl 2005; Ayasse *et al*., 2011). While the chemical basis of the sexual mimicry and the extreme pollinator specificity has been confirmed by field bioassays with synthetic compounds for a growing number of sexually deceptive orchids (see Bohman *et al*., 2016a; Bohman *et al*., 2020a; Peakall *et al*., 2020), these examples represent just a tiny fraction of the hundreds of known cases of orchids employing this pollination strategy (Johnson and Schiestl 2016; Peakall, 2023).

Australia is home to a high proportion of sexually deceptive orchids, where several hundred species spanning 11 genera are now known to use this strategy (Gaskett, 2011; Peakall, 2023). *Cryptostylis* was the first Australian orchid genus discovered to be sexually deceptive (Coleman, 1927), with all five Australian species dependent on the same pollinator, the orchid dupe wasp, *Lissopimpla excelsa* Costa (Ichneumonidae) (Coleman, 1927, 1929, 1930a, 1930b; Nicholls, 1938). While attempted copulation (pseudocopulation) is not always necessary for pollination (Peakall, 2023), *Cryptostylis* represents an extreme amongst sexually deceptive plants as one of only two confirmed cases (the other being the beetle‐pollinated *Disa forficaria* (Cohen *et al*., 2021)) where flowers induce ejaculation by some male pollinators (Coleman, 1930b; Gaskett *et al*., 2008). While it is almost 100 yr since Coleman conducted simple experiments with *Cryptostylis* revealing that wasps could locate hidden flowers, leading to her astute conclusion that scent and mimicry were involved in this case of pollination by sexual deception (Coleman, 1930a), the compounds responsible for pollinator attraction have only just started to be elucidated. In previous experiments with (*S*)‐2‐(tetrahydrofuran‐2‐yl)acetic acid from *Cryptostylis ovata* R.Br, only close approaches by *L. excelsa* have been observed (Bohman *et al*., 2019). As such, it is still unknown what induces attempted copulation in male *L. excelsa*, suggesting that additional chemical cues remain to be discovered.

While the Ichneumonidae is one of the most diverse families of Hymenoptera, with over 25 000 species known (Yu *et al*., 2016), sex pheromones released by females have been structurally elucidated for just three ichneumonid species (Bohman *et al*., 2019). In *Itoplectis conquisitor*, a blend of neral and geranial elicits male sexual activity (Robacker & Hendry, 1977). Eller *et al*. (1984) found that *Syndipnus rubiginosus* uses ethyl (*Z*)‐9‐hexadecenoate as its sex pheromone, while in *Campoletis chlorideae*, tetradecanal and 2‐heptadecanone have been identified as the sex pheromones (Guo *et al*., 2022). Limited data for ichneumonids make it difficult to predict the likely compounds involved in inducing attempted copulation in *L. excelsa* with *Cryptostylis*.

Sex pheromones are typically female‐produced volatile compounds that underpin the chemical sexual communication among conspecifics (Witzgall *et al*., 2010). Some insects, however, produce precursors (pro‐pheromones) that are subsequently modified by external processes to become bioactive compounds. For example, relatively nonvolatile unsaturated long‐chain hydrocarbon pro‐pheromones have been shown to be oxidatively cleaved in air into attractive volatile aldehydes, as demonstrated in sawflies (Bartelt *et al*., 1982, 2002; Bartelt & Jones, 1983; Cossé *et al*., 2002; Staples *et al*., 2009), flies (Collignon, 2011; Lebreton *et al*., 2017), cockroaches (Hatano *et al*., 2020), beetles (Wickham *et al*., 2012) and wasps (Swedenborg & Jones, 1992; Xu *et al*., 2020; Faal *et al*., 2022). Recently, it has also been shown that in poplar and corn, instead of short‐chain aldehydes being directly biosynthesised by the plant, unsaturated waxes are produced that are oxidatively cleaved to yield the bioactive aldehydes, such as nonanal (Chen *et al*., 2023). So far, no evidence of pro‐pheromone mimicry by plants has been presented.

The objective of this study was to investigate whether pro‐pheromone mimicry may be involved in the sexual attraction of the ichneumonid wasp pollinator *L. excelsa* to the orchid *Cryptostylis ovata*, thereby providing the first evidence of the involvement of pro‐pheromone mimicry in pollination.

## Materials and Methods

### Overview of pollination by sexual deception

The degree of sexual behaviour required to achieve pollination varies between sexually deceptive plant species, from pre‐copulatory behaviour only through to prolonged attempted copulation (pseudocopulation) (Peakall, 2023). Despite their remarkable mimicry, a common feature across sexually deceptive plants is that only a fraction of the sexually attracted male insects exhibits the full repertoire of behaviour essential to achieve pollination. This holds true irrespective of whether the pollinators are bees (Schiestl *et al*., 1999), thynnine wasps (Peakall, 1990), ichneumonid wasps (Weinstein *et al*., 2016), fungus gnats (Hayashi *et al*., 2025, 2021), beetles (Cohen *et al*., 2021) or other types of insects. Further, most visitors rapidly develop site‐specific avoidance, which underpins the requirement for the regular movement of bait flowers and chemical baits during field experiments and bioassays (Bohman *et al*., 2020a, 2020b, 2020c).

### Study system

Details of the study system of *Cryptostylis ovata* R.Br. and *L. excelsa* Costa have been summarised previously (Weinstein *et al*., 2016). In short, the genus *Cryptostylis* comprises 25 species, with five species found in Australia. *Cryptostylis ovata* occurs only in high rainfall parts of south‐western Australia. Flowering is between November and March, with the flowers opening sequentially up the scape, such that only one to three flowers are fresh at a given time. Observations of shadehouse plants, where pollinators are excluded, have confirmed that a vector is required for pollination. Pollination is solely by sexually deceived males of the ichneumonid *L. excelsa*. In order to transfer pollen, the pollinators must reverse into the flowers after landing, until the tip of their abdomen contacts the column. As the column is underneath the labellum in some *Cryptostylis* species, including *C. ovata*, this necessitates the wasp moving to the underside of the flower. Unusually for an orchid, the pollinia are attached near the tip of the abdomen.

We knew *a priori* that artificially presented *C. ovata* flowers elicit attempted copulation in a subset of *L. excelsa* male wasp visitors to the flower. For example, at the same study site as the present study, Weinstein *et al*. (2016) reported that on average 33% of landing wasps proceeded to attempted copulation on their first visit to an artificial 1 × 1 m patch of four inflorescences, each with a minimum of two flowers each, during 10 min baiting trials. While *c*. 75% of the visitors immediately left the patch, attempted copulation rates dropped to 22% at second flower visits, and 11% at even rarer third successive flower visits. Unfortunately, data on attempted copulation rates at single bait flowers are unavailable for direct comparisons with the present study.

### Field sites and sources of experimental flowers

**Fig. 1:**
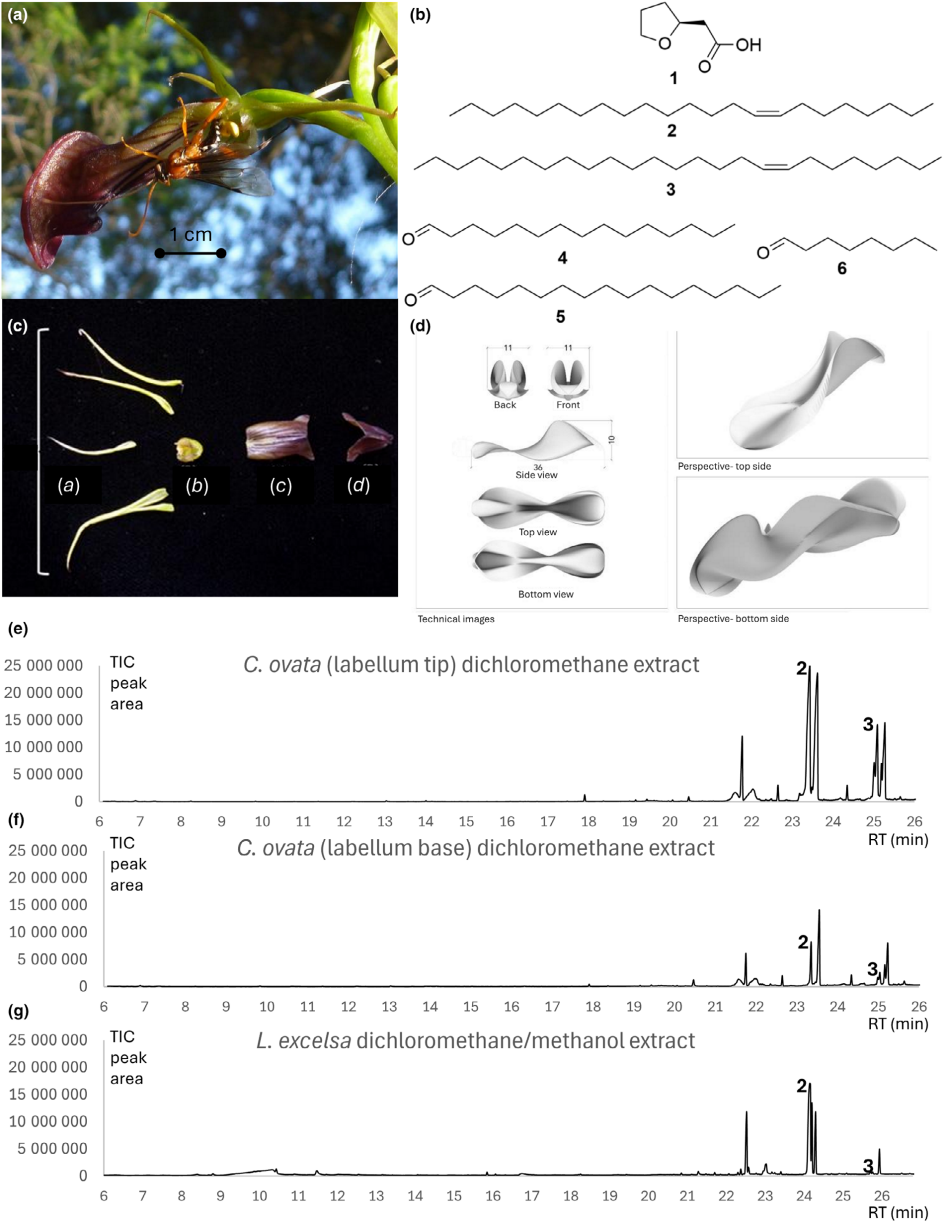
Analysis of plants and pollinators. (a) *Cryptostylis ovata* with its pollinator *Lissopimpla excelsa*, (b) chemical compounds from *C. ovata*, (c) a dissected flower, showing the floral parts used for field trials and extracts; (a) petals and sepals, (b) column and basal portion of labellum, (c) centre of labellum and (d) tip of labellum. (d) Three‐dimensional model of *C. ovata* flower used for bioassays, (e–g) total ion chromatogram (TIC) with retention times (RT) for solvent extracts of *C. ovata* labellum tip, labellum base and abdomen extract of female of pollinator, with compounds **2** and **3** annotated (compounds **1** and **4**–**6** only present at trace levels, not directly visible in the TIC).

*Cryptostylis ovata* flowers (Fig. 1a) were sourced from a population near Capel (33°35′29.69″S, 115°32′31.77″E), and a population near Margaret River (33°58′2.21″S, 115°70′58.37″E). Inflorescences were picked at their base, placed in vials of water and maintained at 4°C until use. All floral presentations were conducted following the baiting method, where flowers of sexually deceptive orchids are moved to a new position in the landscape to renew the response of pollinators (Stoutamire, 1974; Peakall, 1990). All experiments involving flowers were performed at a 600 m × 200 m site within Kings Park and Botanic Garden in Perth, Western Australia (31°57′48.33″S, 115°50′16.80″E). Presentations were conducted between 6:00 am and 12:00 noon during December to coincide with the period of maximum field activity of *L. excelsa* (Tomlinson & Phillips, 2012). Voucher specimens of *C. ovata* are held at the Western Australian Herbarium (voucher number PERTH 06731481).

### Determining the site of odour release

To determine which floral parts are involved in pollinator attraction, flowers were dissected and the separate parts presented to *L. excelsa* males. Preliminary trials confirmed both the petals and the sepals to be comparably attractive; thus, these smaller parts were combined in subsequent trials. Eight flowers, each from different plants, were dissected into four floral parts: A = combined petals and sepals, B = column and basal portion of labellum, C = centre of labellum and D = tip of labellum (Fig. 1b). For each replicate flower, the floral parts were each presented individually in the field, attached to 10‐cm wooden skewers, with each part being exposed in four 3‐min trials. For each presentation, the number of wasps responding to the floral part was recorded, as measured by a sustained approach towards or alighting on the floral part. After data were tested for normality (Shapiro–Wilk normality test, *P* > 0.05) and homogeneity of variances (Levene's test, *P* > 0.05), differences in the number of responses to the floral parts were tested for using a repeated measures ANOVA (*P* < 0.05), followed by pairwise paired *t*‐tests, in R v.3.0.1 (R Core Team, 2013).

### Extraction of floral compounds

To enable the comparison of the chemical profiles of the different floral parts, the separated parts (Fig. 1b) of two flowers were extracted individually: They were crushed individually with a pestle and extracted with dichloromethane (200 μl, 24 h) in an extraction vial (1 ml). As controls, parts of the stems from these flowers were placed in a 1 ml extraction vial and extracted following the same protocol.

### Extraction of insect compounds

Extracts for three female *L. excelsa* were prepared following Bohman *et al*. (2012), by dissecting the wasp into three portions: the head, thorax and abdomen. Each portion was placed in a separate 1‐ml extraction vial and methanolic dichloromethane (1 : 1, *c*. 200 μl) was added over the surface of the structure. The vials were sealed, and tissues extracted for 24 h and stored at −20°C until required.

### Gas chromatography‐mass spectrometry

Gas chromatography‐mass spectrometry (GC‐MS) analysis followed Bohman *et al*. (2019). Electron impact mass spectra (70 eV) were recorded on an Agilent 5973 mass detector connected to an Agilent 6890 gas chromatograph equipped with a BPX5 column ((5% phenyl polysilphenylene‐siloxane), 30 m × 0.25 mm × 0.25 μm film thickness, SGE Australia), using ultra high purity helium as a carrier gas. The scan range was *m/z* 33–600 amu.

### Chemical identification and synthesis

To determine the double‐bond positions of the alkenes, dimethyldisulfide (DMDS) derivatisation of floral extracts and wasp extract was conducted following Bohman *et al*. (2020c). (*Z*)‐Isomers of all alkenes were tentatively assigned based on GC co‐elution of the natural products from extracts and synthetically prepared alkenes. GC co‐elution of epoxides formed from the plant natural products and synthetic alkenes (Ruesch gen. Klaas & Warwel, 1999) supported this assignment.

Synthesis of (*Z*)‐8‐tricosene and (*Z*)‐8‐pentacosene broadly followed Kling *et al*. (1993): Octyltriphenylphosphonium bromide (0.62 g, 1.4 mmol) was dissolved in THF/HMPA 4 : 1 (4 ml) and cooled on an ice bath. Lithium bis(trimethylsilyl)amide (LiHMDS, 1.4 ml, 1.0 M in THF) was added and after 10 min the mixture was cooled on dry ice/acetone and pentadecanal (0.18 g, 0.8 mmol) or heptadecanal (0.20 g, 0.8 mmol) was added and the reaction mixture was stirred at −78°C for 1 h. Saturated ammonium chloride was added, and the product extracted three times with ethyl acetate, washed with water and brine, dried over magnesium sulphate, concentrated *in vacuo* and purified to >97% purity with MPLC (hexanes) to afford 182 mg (70%) (*Z*)‐8‐tricosene and 206 mg (73%) (*Z*)‐8‐pentacosene as white solids.

### Field bioassays of candidate compounds

For field bioassays with thynnine wasps (Thynnidae), which have large‐winged males and smaller wingless females, a 4‐mm‐diameter black bead has been widely used as the model (Peakall *et al*., 2020). However, the winged females of *L. excelsa* are similar in size to the male pollinators of *C. ovata* (males 15–30 mm total body length). Therefore, we used a 3D plastic model of a *C. ovata* flower to provide the male wasps with a more realistically sized structure on which to land. The model was designed in the rhinoceros v.6 3D computer‐aided design application software. The dimensions (Fig. 1d) were based on the average length and width of healthy‐looking *C. ovata* flowers, collected during their peak flowering season. The ‘flower dummies’ were printed in red‐coloured poly lactic acid (PLA) on a Creality CR‐10 3D printer with a 0.4 mm nozzle size as a close approximation to the predominantly red‐coloured females and flowers.

Given that the male *L. excelsa* wasp pollinators were at low density in the landscape and that the intensity of sexual behaviour appears to vary considerably between days, a modified consecutive single bioassay approach was utilised in this study (Bohman *et al*., 2016b, 2020b). Each day, a ‘baseline control’ synthetic blend consisting of compounds **1**–**3**, which had previously been confirmed to be sexually attractive, and a test ‘treatment’ synthetic blend were applied to new flower dummies. This ‘baseline control’ provided the advantage of constancy over using an inflorescence of *C. ovata*, as they vary in the number of flowers open at a given time. Experiments involved alternating 3‐min‐long presentations of either the control or the treatment, with each baiting trial conducted at least 10 m from the previous baiting location to renew the pollinator response (Peakall, 1990). Each experiment was repeated in total 12 times over at least 2 d.

As has been common practice across studies investigating the chemistry of pollinator attraction in Australian sexually deceptive orchids (Bohman *et al*., 2020a, 2020b, 2020c), pollinator responses were scored in three mutually exclusive categories: *A* = approach only (targeted flight towards the dummy) without landing, *L* = landing on the dummy only, *C* = attempted copulation after landing on the dummy. Attempted copulation was recognized based on repeated probing of the model with the tip of the abdomen.

The bioassays were conducted at three sites in suburban Perth: Mosman Park (32°01′02.3″S 115°45′18.0″E), Kings Park and Botanic Garden (31°57′44.5″S 115°50′18.5″E) and Warnbro (32°19′37.0″S 115°46′19.9″E), where *L. excelsa* occurs in suitable numbers for experiments (Weinstein *et al*., 2016; Bohman *et al*., 2019). All field bioassays with candidate compounds were conducted between 7 and 10 am, over the period 15 December to 21 December 2023, and for experiments with compound **1** alone, 23 November to 28 November 2024.

### Statistical analysis of field bioassays

R v.4.4.0 (2024‐04‐24) was used for visualisation and statistical analysis (R Core Team, 2024) and included the use of the packages ggplot2, ggpubr, rstatix and stats within rstudio (Posit Software, PBC). As in some other studies involving field bioassays with female dummies (e.g. Bohman *et al*., 2020b), given zero to low rates of attempted copulation (*C*) in some treatments, our statistical analysis considered either: (1) the ‘Total’ wasp responses, which consists of the sum of the mutually exclusive *A*, *L* and *C* counts per trial; or (2) the ‘*LC*’ wasp response, as *L* + *C* counts are deemed to collectively represent a stronger sexual response at the dummy than mere approaches. Finally, we estimated percent attempted copulation %*C* as *C*/LC*100 (Weinstein *et al*., 2016).

We assumed that each observation (*A*, *L* or *C*) was independent and did not include revisitation within trials. As in previous studies (reviewed by Bohman *et al*., 2020a), we deem this to be a reasonable assumption given the short 3‐min duration of the trials and that multiple wasp individuals often visit the experimental dummies simultaneously. Significant variation in the total counts per trial was found over the 7‐d study window for the control blend (Kruskal–Wallis, *P* = 0.002). Therefore, we restricted formal statistical analysis to replicate experiments that were conducted over two consecutive days. Further, given the modest sample sizes, we applied non‐parametric two‐sample Wilcoxon tests.

## Results and Discussion

After demonstrating that (*S*)‐2‐(tetrahydrofuran‐2‐yl)acetic acid derivatives constitute attractants that *C. ovata* uses to lure male *L. excelsa* as pollinators (Bohman *et al*., 2019), we have been working to discover the missing compound(s) required to elicit the pseudocopulatory behaviour that the pollinators frequently exhibit at the flowers. To shorten the list of candidate floral volatiles from whole flowers, we dissected *C. ovata* flowers into four sections (Fig. 1c), which were tested individually in field bioassays to see which parts were most attractive to pollinators. In total, 382 wasp responses were recorded, with average responses per trial to the centre (3.53 ± 0.43 SE) and tip (4.18 ± 0.53 SE) of the labellum being significantly higher (ANOVA: *P* < 0.01, pairwise *t‐*tests: *P* < 0.05) than to the labellum base (1.82 ± 0.36 SE) and sepals/petals (1.71 ± 0.30 SE). Furthermore, attempted copulation was observed at both the large centre and tip sections of the labellum, which contrasts with most other sexually deceptive orchids (Perkins *et al*., 2023), where the attraction is pinpointed to small sections of the flower. For example, among the Australian species, the small callus structure of the labellum is the attractive tissue in *Chiloglottis* (de Jager & Peakall, 2016) and *Drakaea* (Phillips *et al*., 2013), while the glandular sepal tips are the source of attraction in many *Caladenia* (Phillips *et al*., 2024).

The chemical compositions of the various floral parts were analysed with GC‐MS, and the components of the more attractive floral tissue were compared with those of less attractive floral tissue. We detected C_23_‐ and C_25_‐unsaturated hydrocarbons in the attractive floral tissue (Fig. 1e). Meanwhile, in females of *L. excelsa*, C_23_‐unsaturated hydrocarbons represented the largest peak in the solvent extracts of all dissections, whereas only traces of C_25_‐alkenes were detected. After DMDS derivatisation to determine alkene positional isomers (Bohman *et al*., 2020c), GC‐MS analysis revealed that two main compounds, 8‐tricosene (characteristic fragment ions of *m/z* = 159/257) and 8‐pentacosene (characteristic fragment ions of *m/z* = 159/285), were present in the flowers, with traces of 10‐pentacosene (characteristic fragment ions of *m*/*z* = 187/257). In female *L. excelsa*, 8‐tricosene was the prominent alkene, with a minor second alkene identified as 6‐tricosene (characteristic fragment ions of *m*/*z* = 131/285). Therefore, (*Z*)‐8‐Tricosene (**2**) and (*Z*)‐8‐pentacosene (**3**) were synthesised and, together with the previously identified (*S*)‐2‐(tetrahydrofuran‐2‐yl)acetic acid (**1**) (Fig. 1b), formulated into blends for field bioassays.

Consistent with our previous findings (Bohman *et al*., 2019), tests with the tetrahydrofuran acid **1** only, on 3D plastic models of *C. ovata* flowers, elicited rapid approaches (*A*) from male wasps, but only one landing (*L*) and no attempted copulation (*C*) at the spiked dummy (**1** only: *A* = 21, *LC* = 1, %*C* = 0, 12 trials over 3 d). By contrast, in preliminary experiments involving **1** and the alkenes **2** and **3**, in ratios of **1** : **2** : **3** varying from 1 : 10 : 5 (in total *c*. 0.2 mg), 1 : 100 : 50 (in total *c*. 2 mg) to 10 : 100 : 50 (in total *c*. 2 mg), landings (*L*) were induced and one attempted copulation (*C*) was observed (combined results: *A* = 124, *LC* = 18, %*C* = 6, 36 trials over 4 d). Further preliminary tests over 2 d, with threefold more concentrated alkenes, **2** and **3**, indicated increased landings and attempted copulation (**1 : 2 : 3** at 3 : 300 : 150: *A* = 45, *LC* = 33, %*C* = 27, 18 trials over 2 d). Over this same period, a negative control, consisting of dummies with only solvent, did not induce any approaches, landings or attempted copulation (12 trials over 2 d). In the light of these preliminary results, a 3 : 300 : 150 blend of tetrahydrofuran acid and alkene compounds **1 : 2 : 3** was established as the baseline control blend in subsequent experiments.

Although similar mono‐unsaturated long‐chain hydrocarbons are pollinator attractants in some sexually deceptive *Ophrys* orchids (Schiestl *et al*., 1999, 2000, 2003; Stökl *et al*., 2005), the very large amounts required to induce frequent sexual behaviour of *L. excelsa* led us to conclude that these rather nonvolatile compounds were unlikely to be the active compounds. Therefore, we hypothesised that these alkenes, present primarily in the active floral tissue, might be precursors to other, more volatile sexual attractants. Apart from the requirement of unusually large amounts of alkenes for activity, this hypothesis was also based on: (1) previous discoveries of aldehydes as sex pheromones from other parasitic wasps, which are proposed to be products of oxidative cleavage of unsaturated cuticular hydrocarbons (Swedenborg & Jones, 1992; Xu *et al*., 2020); (2) our observation that the pollinator‐attractive parts of *C. ovata* make up an unusually large area of the flower for a sexually deceptive orchid (Fig. 1a,c), potentially providing a relatively large surface area where oxidative cleavage can take place; and (3) that the alkenes are uncommon in nature, with double bonds located on even‐numbered carbons, potentially giving rise to unusual aldehydes.

In a targeted analysis for the putative aldehyde products from our identified alkenes, proposed to be pentadecanal (**4**), heptadecanal (**5**) and octanal (**6**), we found small amounts of compound **4** and traces of **5** in the attractive floral tissues, although we were unable to detect any octanal (**6**). In the solvent extracts of females of *L. excelsa* (*n* = 3), traces of all aldehydes **4**–**6** were found, thus warranting the testing of these aldehydes in combination with **1** for pollinator attraction.

**Fig. 2:**
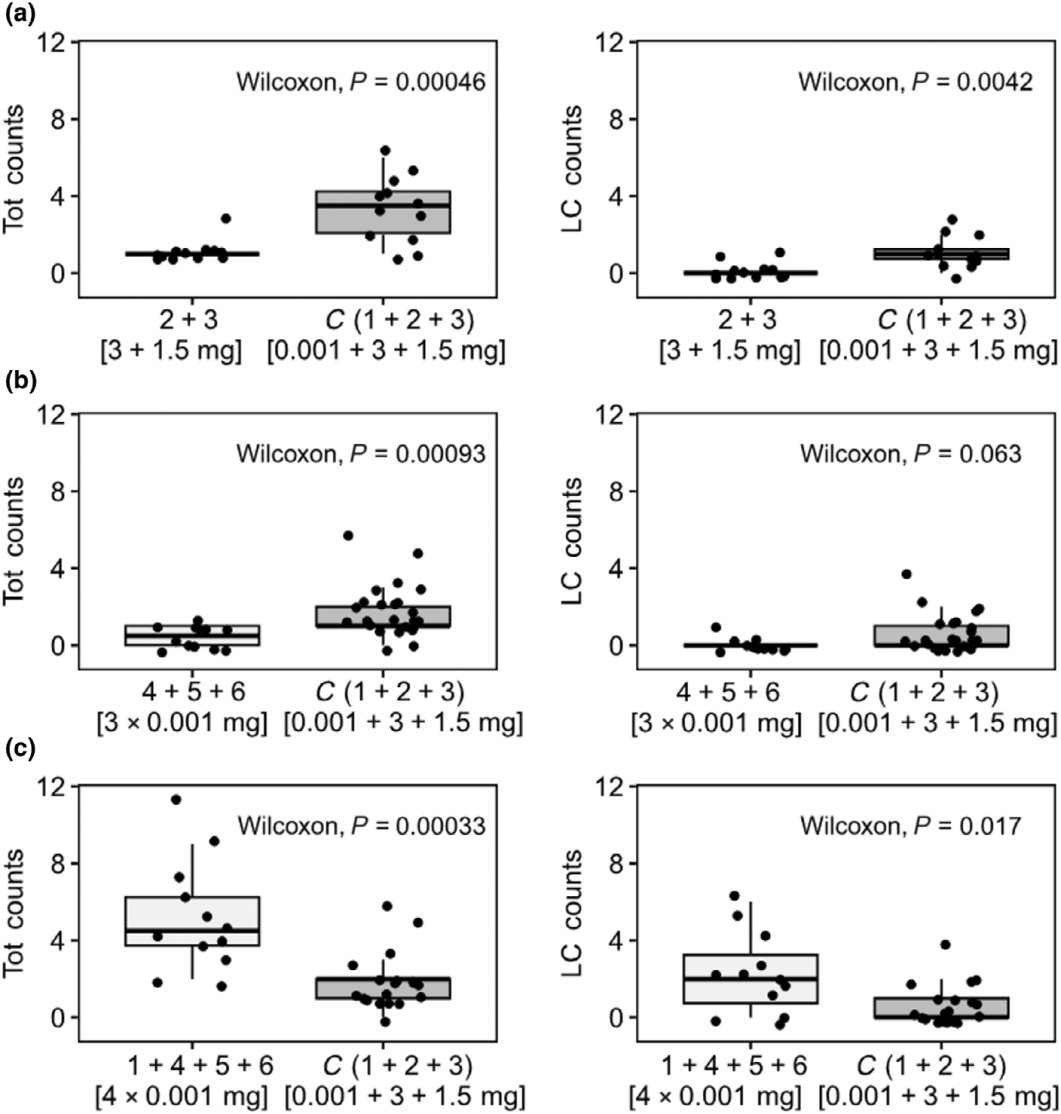
Boxplots summarising the responses of male *Lissopimpla excelsa* wasps to bioassays presenting choices of compounds **1**–**6** in different combinations and at different amounts. The left panels show responses as total counts (*A* + *L* + *C*) per trial (*A* = wasp approaches only, *L* = wasp approach then landing, *C* = attempted copulation after landing), while the right panels show responses of *LC* counts (*L* + *C*), deemed to represent a stronger sexual response than approaches. Outcomes of Wilcoxon tests of the null hypothesis of no difference in the count distributions between control and treatment are also shown. (a) Treatment compounds **2** + **3** vs baseline control **1** + **2** + **3**, each at total amounts of 4.5 mg. (b) Treatment compounds **4** + **5** + **6** vs baseline control **1** + **2** + **3**, at total amounts of 0.003 and 4.5 mg, respectively. (c) Treatment compounds **1** + **4** + **5** + **6** vs baseline control **1** + **2** + **3**, at total amounts of 0.004 mg and 4.5 mg, respectively. Median and quartiles shown in the boxplots were calculated over replicate experiments on consecutive days.

The combination of **1** and the aldehydes **4**–**6** (*c*. 0.003 mg), despite 1000‐fold lower levels of the aldehydes relative to the alkenes, elicited both significantly higher total counts per trial and significantly more *LC* counts than the baseline control blend (Fig. 2c; Supporting Information Video S1: video of copulatory behaviour on the dummies; **1, 4–6**: A = 19, *LC* = 27, %*C* = 29.6, 12 trials over 2 d, **1 : 2 : 3** at 3 : 300 : 150, A = 23, *LC* = 14, %*C* = 14.3, 19 trials over 2 d, Fig. 2c). Furthermore, the **1, 4–6** blend yielded the highest percentage of attempted copulation observed across the study (29%).

When **1** was removed from the blend with alkenes **2** and **3**, there were significantly fewer total and *LC* counts per trial, and no attempted copulation (**2 : 3** at 300 : 150, *A* = 12, *LC* = 2, %*C* = 0, 12 trials over 2 d, **1 : 2 : 3** at 3 : 300 : 150, *A* = 27, *LC* = 13, %*C* = 23, 12 trials over 2 d, Fig. 2a). Similarly, when compound **1** was removed from a blend of the aldehydes **4**–**6**, there were significantly fewer total counts per trial than the baseline control (**4–6**: *A* = 5, *LC* = 1, %*C* = 0, 12 trials over 2 d, **1 : 2 : 3** at 3 : 300 : 150, *A* = 28, *LC* = 15, %*C* = 13.3, 24 trials over 2 d, Fig. 2b).

To summarise our results, the tetrahydrofuran acid **1** on its own rapidly elicits approaches but rarely lands, and no attempted copulation. In the absence of **1**, total wasp responses were significantly reduced in experiments with alkenes **2** and **3** only (Fig. 2a) and the aldehydes **4–6** only (Fig. 2b). These findings indicate a key role of the acid **1** in longer‐range pollinator attraction, as previously predicted (Bohman *et al*., 2019). The blend eliciting the strongest attraction (total count), strongest sexual response (*LC* counts) and the highest observed attempted copulation of 29.6% was the combination of the acid **1** with small amounts of the aldehydes **4–6** (Fig. 2c). While the percentage of wasps engaging in attempted copulation with our synthetic blend of (**1** + **4**–**6**) was close to the maximum of 33% reported at patches of multiple inflorescences (Weinstein *et al*., 2016), attempted copulation rates might be enhanced at the flower by additional visual and physical cues.

Overall, despite the alkenes **2** and **3** being dominant peaks in the floral and wasp extracts (Fig. 1e–g), the required amounts to achieve pollinator attraction was unnaturally high, even when allowing for their low volatility. However, the aldehydes **4**–**6**, derived from **2** and **3**, were present in trace amounts in the female wasps, but were far more attractive in bioassays and induced attempted copulation at 1000‐fold lower quantities than the alkenes in the baseline blend. A plausible explanation is that **2** and **3** are pro‐pheromones for *L. excelsa*, which are oxidatively cleaved to produce the aldehydes **4**–**6** as the active attractants, alongside **1**. Given the abundance of **2** and **3** in *C. ovata* flowers, we suggest this may be the first known example of a pro‐pheromone mimicry pollination system, in this case involving the ‘passive’ oxidation of alkenes to aldehydes. Given that all five Australian *Cryptostylis* species use the same male wasp pollinator, *L. excelsa*, we expect this strategy will apply to all of them.

To test the pro‐pheromone mimicry hypothesis in *Cryptostylis* and in sexually deceptive orchids more broadly, it will be imperative to analyse flowers from different developmental stages and ages, as exposure to heat, UV radiation and other environmental factors would affect the ratio of alkenes and aldehydes. Application of antioxidants on flowers, similar to what has previously been experimented with for insect pro‐pheromones (Bartelt & Jones, 1983), and transcriptomic studies aiming to elucidate the biosynthetic pathways in *C. ovata* (e.g. Xu *et al*., 2017), are needed to further test this hypothesis. In particular, transcriptomic studies could provide evidence of whether or not any or all aldehydes are biosynthesised by the flower. To detect other cases of possible pro‐pheromone mimicry in sexually deceptive orchids, it may be worth re‐opening investigations into orchids that use alkenes as pollinator attractants and/or have low rates of attempted copulation in bioassays.

To test whether orchid pollination systems suggested to utilize pro‐pheromone mimicry operate in a similar fashion to other sexually deceptive orchids, requirements for structural specificity of the alkenes and aldehydes should be investigated. It is noteworthy that the alkenes in *C. ovata* and *L. excelsa* have the unusual position of the double bonds on even‐numbered carbons, giving rise to the uncommon aldehydes pentadecanal and heptadecanal. Structurally specific and/or unusual aldehydes may be required to achieve the specific communication with the targeted pollinator. Alternatively, specificity could also rely on the uniqueness of other attractants, such as **1**, acting in concert with aldehydes more broadly.

Oxidative cleavage of cuticular wax alkenes to aldehydes in plant leaves was only discovered very recently (Chen *et al*., 2023). Here, we show that a sexually deceptive plant may also rely on this mechanism for pollinator attraction. Pro‐pheromones and pro‐pheromone mimicry may actually be major, but often overlooked, strategies of mate attraction in insects (Bartelt *et al*., 2002; Hatano *et al*., 2020) and the plants that mimic them, particularly since many aldehydes have extremely low detection thresholds by their receivers (Lebreton *et al*., 2017; Becher *et al*., 2018), allowing these compounds to operate at very low concentrations, thereby going undetectable in routine analysis.

## Competing interests

None declared.

## Author contributions

RP, RDP and BB conceptualised the study; BB, SvK, AMW and BO collected the data; RP, BB, AMW and GRF analysed the data. BB and GRF synthesised and confirmed chemical structures. BB and RDP wrote the first version of the manuscript. All authors edited the manuscript.

## Supporting information


**Dataset S1** GC‐MS files of *Cryptostylis ovata* and *Lissopimpla excelsa* extracts.

**Video S1** Video of the behaviour of *Lissopimpla excelsa* on the 3D model of *Cryptostylis ovata*, treated with a blend of synthetic attractants **1** + **4** + **5** + **6**.
Please note: Wiley is not responsible for the content or functionality of any Supporting Information supplied by the authors. Any queries (other than missing material) should be directed to the *New Phytologist* Central Office.

## Data Availability

The data that support the findings of this study are available in the Supporting Information of this article (Dataset S1 contains raw GC‐MS data for Fig. 1).
